# Amnion-Derived Multipotent Progenitor Cells Improve Achilles Tendon Repair in Rats

**Published:** 2013-06-19

**Authors:** Justin Philip, Florian Hackl, José A. Canseco, Rami A. Kamel, Elizabeth Kiwanuka, Jesus Rodrigo Diaz-Siso, Edward J. Caterson, Johan P. E. Junker, Elof Eriksson

**Affiliations:** ^a^Laboratory for Tissue Repair and Gene Therapy, Division of Plastic Surgery; ^b^Laboratory for Tissue Engineering and Regenerative Medicine, Department of Anesthesiology, Brigham and Women's Hospital, Harvard Medical School, Boston, Mass; ^c^Harvard-MIT Division of Health Sciences and Technology, Massachusetts Institute of Technology, Cambridge

## Abstract

**Objective:** Tendon injuries produce considerable morbidity, long-lasting disability, and remain a considerable challenge for clinicians and patients. The objective of the study was to assess the effect of amnion-derived multipotent progenitor (AMP) cells and amnion-derived cell cytokine solution on Achilles tendon healing by using a rat model. **Methods:** Achilles tendons of Sprague-Dawley rats were exposed and transected. The distal and proximal ends were injected with either saline, amnion-derived cell cytokine solution, or AMP cells in a standardized fashion and then sutured by using a Kessler technique. Tendons from each group (n = 6-13) were collected at weeks 1, 2, and 4 postoperatively and assessed for material properties (ultimate tensile strength, Young modulus, yield strength, and breaking strength). Tendons were also evaluated histologically for cross-sectional area by using hematoxylin-eosin and trichrome stains. **Results:** Mechanical testing showed that the Young modulus was significantly higher in AMP cells–treated tendons at week 4 compared with both saline-treated and amnion-derived cell cytokine solution–treated tendons. Yield strength was significantly higher in the AMP cells–treated group compared with saline-treated controls at week 4. No significant differences were observed between the study groups at weeks 1 and 2. **Discussion:** Amnion-derived multipotent progenitor cells have a positive effect on healing tendons by improving mechanical strength and elastic modulus during the healing process. The presented findings suggest the clinical utility of AMP cells in facilitating the healing of ruptured tendons. Both the Young modulus and yield strengths of tendons increased significantly following treatment with AMP cells.

The healing of tendon injuries such as tears or lacerations represent a considerable challenge.^1^ Tendon healing is a slow process, lasting for months, that aims to reestablish tendon fiber continuity and strength.^2^ The tendon healing process occurs in 3 phases starting with the inflammatory phase, that is characterized by proinflammatory molecules and numerous cytokines and growth factors, recruiting fibroblasts and local tenocytes.^2^ In the proliferative phase, the cells begin to synthesize the extracellular matrix, primarily type III collagen. Finally, the tendon enters the remodeling phase, where the initial type III collagen is replaced by collagen type I and organized longitudinally along the tendon axis over a long period of time.^3^ However, tendon healing frequently results in the formation of scars that reduce functionality.^3^

Modalities that speed up the healing process present a great clinical value. Growth factors have shown to play an essential role in mediating the tendon healing process.^4^^,^^5^ Vascular endothelial growth factor,^6^ platelet-derived growth factor,^7^ and transforming growth factor,^7^^,^^8^ among others, have been shown to be markedly upregulated following tendon injury and are active at multiple stages of the healing process.

Stem cells and stem cell-like multipotent cells are defined by their ability to differentiate into several different cell types.^9^^,^^10^ They are also known to produce cytokine growth factors that serve as mediators to the cellular processes of the wound-healing cascade, thus enhancing regeneration.^11^^,^^12^ Amnion-derived cells share numerous characteristics with stem cells, including differentiation potential.^13^^,^^14^ They also have been shown to secrete a number of cytokines and growth factors^15^ essential to tendon regeneration. In previous studies, amnion-derived multipotent progenitor (AMP) cells have shown a positive effect on mechanical properties in an acute healing model in abdominal fascia in rats.^16^

The hypothesis of this study was that the rate of tendon repair and the mechanical properties after tendon injury can be improved by intratendinous injection of cultured human AMP cells and amnion derived cellular cytokine solution (ACCS).

## METHODS

### Cell and cytokine solution preparation

Amnion-derived multipotent progenitor cells were provided by Stemnion Inc (Pittsburgh, Pa). Amnion cells were harvested from human placentas delivered at the time of caesarean delivery. The cells were transported frozen and thawed immediately before experimental application.

Amnion-derived multipotent progenitor cells were grown to confluence, and the supernatant was harvested.^11^ The secreted product, labeled ACCS, was used as one of the treatment modalities in the study. Detailed analysis of the secreted growth factor and cytokine profile, including, but not limited to, platelet-derived growth factor-BB, vascular endothelial growth factor, angiogenin, tissue inhibitor of metalloproteinase-1, and tissue inhibitor of metalloproteinase-2, has previously been reported.^11^

### Animals

One hundred twenty-six female Sprague-Dawley rats (Charles River, Cambridge, Mass), weighing approximately 300 g and 10 weeks old, were used in this experiment. Two animals were kept per cage and given food and water ad libitum. Each cage (n = 63) was randomly assigned to 3 different groups: saline (control), ACCS, and AMP cells. The study was approved by the Harvard Medical Area standing committee on animals (protocol #04794), and all institutional guidelines for the care and treatment of laboratory animals were followed.

### Surgical procedure

**Figure 1:**
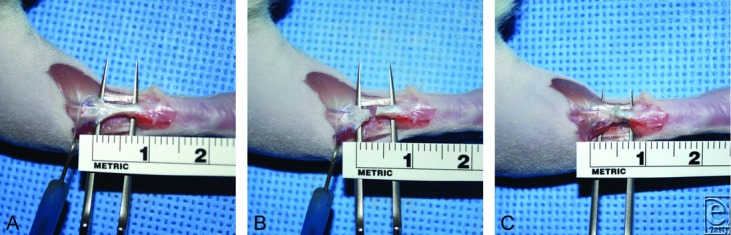
Achilles tendon dissection and repair. (*a*) Sharp dissection was used to expose the Achilles tendon. (*b*) Achilles tendon transection. 100 μL of amnion-derived cell cytokine solution, amnion-derived multipotent progenitor cells, or saline (control) were injected in both proximal and distal ends of the tendon. (*c*) The tendon was approximated, using a Kessler technique with a 6-0 braided suture.

**Figure 2:**
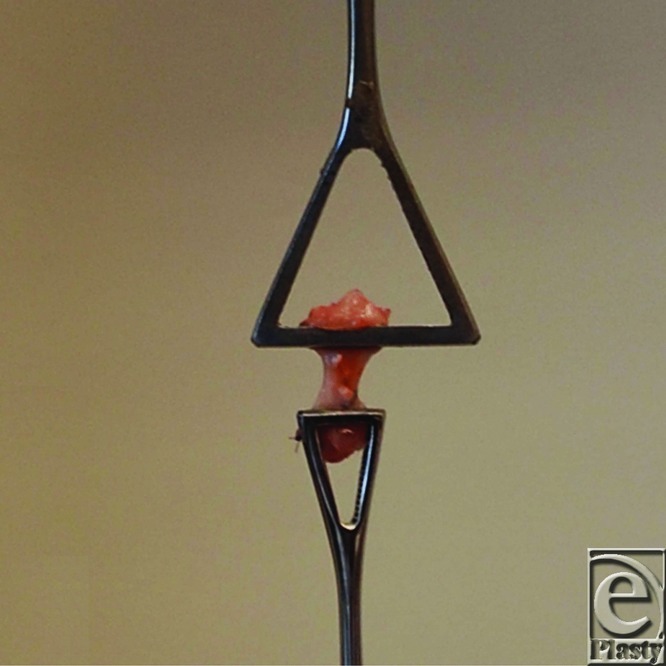
Tensile testing setup. After harvest of the tendons, the distal and proximal ends of each specimen were gripped, using Pennington clamps. The Pennington clamps were secured in the materials testing machine by using pneumatic grips.

Rats were anesthetized with ketamine (60 mg/kg intraperitoneal; Phoenix, St Joseph, Mo) and xylazine (10 mg/kg intraperitoneal; Vedco INC, St Joseph, Mo) and maintained by using isoflurane gas (1%-2%) via nose cone. The right hind leg was shaved and then sterilized by using 70% alcohol, betadine, and 70% alcohol sequentially. Using sharp dissection, the Achilles tendon was exposed and transected at its midpoint (Fig 1). The distal and proximal ends were injected with either 100-μL saline, 100-μL ACCS, or 100 000 AMP cells diluted in 100-μL phosphate-buffered saline. The ends were proximated and repaired by using a modified Kessler technique^17^^,^^18^ with 6-0 ethylene-braided sutures (Ethicon, Somerville, NJ) (Fig 2). Skin was closed, using 6-0 nylon sutures (Ethicon). The right hind limb was then wrapped in petroleum gauze and then immobilized by using a casting tape (Scotchcast™Plus (3M, St. Paul, MN)) that was applied from the toes to the abdomen, achieving 3-point stability (ankle-knee-hip).^19^ The animals were then returned to their cage and allowed to heal for either 1, 2, or 4 weeks. Rats were observed daily for signs of appetite, pain, infection, swelling, and muscle paralysis. Rats were given buprenex (0.05 mg/kg subcutaneous; Reckitt Benckiser Healthcare, Hull, England, United Kingdom) postoperatively for up to 48 hours. All casts were removed 1-week postprocedure to allow weight-bearing.^20^

**Figure 3:**
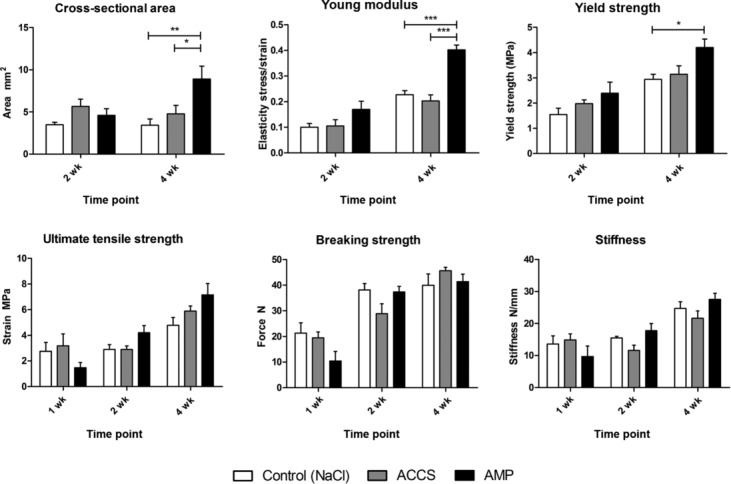
Results of mechanical testing. The results of the mechanical testing are presented in regard to the following parameters: ultimate tensile strength (maximum stress or force per unit area), Young modulus (resistance to elastic deformation), yield strength (maximum stress in elastic region), cross-sectional area, breaking strength (maximum force), and stiffness (force required per unit displacement). ACCS indicates amnion-derived cell cytokine solution; AMP, amnion-derived multipotent progenitor cells.

Animals were euthanized by isoflurane overdose (10%), and Achilles tendons were dissected free from the extraneous soft tissue and harvested together with the calcaneal bone and parts of the gastrocnemius and soleus muscle complex. The mechanical testing specimens were immediately frozen at −80°C. Specimens for histology were dissected in a similar manner without the calcaneus. Contralateral uninjured tendons from each animal were harvested as controls (Fig 3).

### Mechanical testing

**Figure 4:**
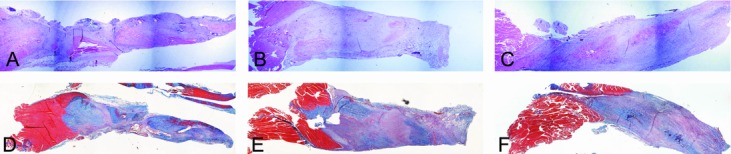
Histological evaluation 2 weeks after tendon repair. Hematoxylin-eosin staining of tendons injected with saline (*a*), amnion-derived cell cytokine solution (*b*), and amnion-derived multipotent progenitor cells (*c*) 2 weeks after repair. Corresponding serial sections stained with Masson trichrome (*d*-*f*).

On the day of evaluation, specimens were thawed to room temperature and prepared for mechanical testing. The muscle was carefully separated from the proximal tendon by blunt dissection to produce a fan of tendon fibers^21^ that were then gripped by using a large Pennington clamp (Johnson & Johnson, New Brunswick, NJ). The distal end of the tendon was then gripped by using another Pennington clamp proximal to the calcaneal insertion (Fig 4). The 2 Pennington clamps were then vertically secured in an Instron 5565 material testing system (Instron, Norwood, Mass) by using pneumatic grips with serrated jaw faces. Tendon width, thickness, and length (distance between Pennington clamp ends) were recorded by using a slide caliper.^21^ The cross-sectional area was calculated, assuming circular tendon geometry. Tendons were kept moist by using gauze with saline throughout testing.

Tendons were preconditioned for 3 cycles at 2% extension immediately followed by a load-to-failure test at a speed of 6 mm/min.^22^ Loading force was measured by using a 100-N load cell, and all data were collected with Blue-Hill 2 software (Instron). *Breaking strength* (defined as maximum force), *stiffness* (force required per unit displacement), *tensile strain* (defined as change in length over initial length mm/mm), *ultimate tensile strength* (defined as maximum stress or force per unit area), yield strength (defined as maximum stress in elastic region), and *Young modulus* (a measure of a material's resistance to elastic deformation) were obtained, and engineering stress versus engineering strain curves generated.

### Histology

The tendons were immersed in 10% formalin for 24 hours and then rinsed in phosphate-buffered saline. Tendons were then cut longitudinally for histological evaluation. Paraffin-embedded sections were mounted onto slides and stained with hematoxylin-eosin and Masson trichrome, using standard protocols. An average of 3 transverse measurements taken at the midpoint, proximal end, and distal end was used to calculate tendon diameter and transverse cross-sectional area.

### Statistical analysis

Data were subjected to 2-way analysis of variance, with treatment and time as independent factors. A Bonferroni post hoc test was used to determine statistical significance. A *P* value < .05 was considered statistically significant. All values are given as mean±SD.

## RESULTS

### Material properties

One hundred twenty-six rat Achilles tendons were transected, injected with saline, ACCS, or AMP cells, then sutured, and allowed to heal for 1, 2, or 4 weeks before being euthanized. Overall, the surgical procedure was well tolerated, with a total of 2 rats (1.5%) lost postoperatively. No discernible differences in any material properties were observed between specimens at the 1-week time point. Because of the fragile nature of the healing tendons, especially at the 1-week time point, tendons that showed incomplete healing due to rupture were excluded (n = 30).

#### Ultimate tensile strength

The AMP cells–treated tendons demonstrated higher ultimate tensile strength at both weeks 2 (4.2±1.68 MPa) and 4 (7.16±2.16 MPa) than saline-treated controls (2.9±0.54 MPa and 4.78±1.63 MPa, respectively). These differences were not statistically significant.

#### Young modulus

The AMP cells–treated tendons exhibited significantly higher (*P* < .005) Young modulus at week 4 (0.25±0.02 MPa) than saline-treated (0.09±0.04 MPa) and ACCS-treated (0.12±0.020 MPa) tendons at the same time point. Uninjured tendons were found to have a Young modulus of 0.33±0.16 MPa.

#### Yield strength

The yield strength of AMP cells–treated tendons (4.2±0.83 MPa) was also significantly higher (*P* < .05) than saline-treated controls (2.93±0.54 MPa) at week 4.

#### Breaking strength and stiffness

As expected, the maximum force (breaking strength) sustained by the tendon specimens as well as tendon stiffness increased with time; however, no statistical significance was seen between treatment groups.

A summary of all measured parameters is given in Table 1.

### Histology

**Figure 5:**
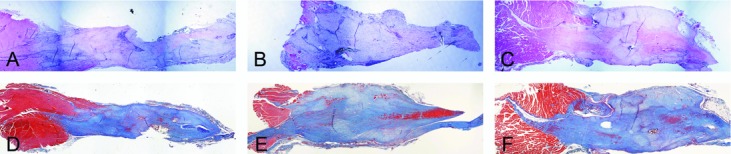
Histological evaluation 4 weeks after tendon repair. Hematoxylin-eosin staining of tendons injected with saline (*a*), amnion-derived cell cytokine solution (*b*), and amnion-derived multipotent progenitor cells (*c*) 4 weeks after repair. Corresponding serial sections stained with Masson trichrome (*d*-*f*).

Histological examination revealed noticeable gapping at the site of repair 1-week postprocedure (data not shown). The AMP cells– and ACCS-treated tendons were visibly thicker at time points 2 and 4 weeks (Figs 4 and 5). The trichrome staining reveals a relative increase in amount of collagen in samples 4-week postprocedure, most apparent in tendons treated with AMP cells (Fig 5).

### Cross sectional area

Both AMP cells– and ACCS-treated tendons showed a significant (*P* < .01 and *P* < .05, respectively) increase in cross-sectional area compared with saline-treated controls 4-week postprocedure (Fig 3).

## DISCUSSION

The results obtained in this study reveal that treatment of transected tendons with AMP cells has a beneficial effect in regard to the material properties of the healed tendons at 4 weeks. The results obtained in this study are similar to those reported previously by Eliasson et al.^23^

A study by Clavert et al 24 concludes that freezing and thawing of human tendons had no influence on the tendinous relaxation but altered significantly the ultimate tensile failure and Young modulus of the tendons. However, a recent study in a porcine model suggests that freezing and thawing of tendons does not alter their tensile properties in a cyclic loading model.^25^ Another recent study in a cadaveric human tendon model has proven that fewer than 3 cycles of freezing and thawing did not influence biomechanical properties.^26^ In this study, any effect attributed to freezing and thawing of tendons is equal for all study groups and hence would not alter the results.

Significant differences were found in the measurements of yield strength and elastic modulus.

Yield strength is the maximum stress a material can sustain before being plastically (permanently) deformed. Thus, in a physical sense, yield strength marks the upper threshold to which a tendon can be stressed. Higher yield strength of a tendon allows for a higher energy dissipation capacity. The tendons treated with AMP cells display significantly higher yield strength 4-week postprocedure than the control group.

Young modulus is a measure of the relative stiffness/elastic modulus of a material. The closer the Young modulus of a healed tendon is to that of a normal, uninjured tendon, the more similarly it will behave under mechanical stress. Excessive stiffness (by 2 or more orders of magnitude) over normal is not desirable as the tendons will display a diminished ability to withstand deformations under stress. In this experiment, the tendons treated with AMP cells and allowed to heal for 4 weeks exhibited a significant improvement in elastic modulus over both saline-treated and ACCS-treated tendons at the same time point. In fact, AMP cells–treated tendons were very close to values observed in untreated tendon by this measurement. Even at the 2-week time point, a trend in the same direction was observed.

Ultimate tensile strength is a measure of the maximum stress that a material can withstand while being subjected to stress. In this experiment, a trend for improved strength was observed with the AMP cells–treated tendons, starting at the 2-week time point and continuing to improve at the 4-week time point. However, this positive trend was not statistically significant.

The greater the tendon cross-sectional area, the greater the amount of new tissue generated. This is an encouraging observation, particularly during the early phase of the healing process as it provides better tendon strength. Hematoxylin-eosin and trichrome stains of tendon samples revealed that the AMP cells-treated tendons at the 4-week time point had a considerably greater transverse cross-sectional area than either saline- or ACCS-treated tendons.

Breaking strength measures the ability of a material to resist breaking or rupture from a tension force. In these experiments, breaking strength did not exhibit any discernible difference. However, at 2 weeks, more tissues had healed to the point where they could undergo analysis by tension testing in both the ACCS- and AMP cells–treated groups, suggesting that there was an earlier improvement in healing when treated with ACCS and AMP cells as compared with saline.

The lack of improved healing of the ACCS-treated tendons may be explained by the fact that the treatment was only performed once. The half-life of the cytokines in tissue was not studied in this experimental setup, but the effects of enzymatic degradation are apparent. This can also explain the positive healing outcome after treatment with AMP cells. The cells likely remain present at the healing site and, in addition to any direct effect on tissue remodeling, could produce active molecules that recruit endogenous cells that improve the tendon healing. The immune privilege of human amnion-derived cells allowed us to apply human AMP cells as a xenoraft in this rat model.^27^

## CONCLUSIONS

This study demonstrates that tendons treated with AMP cells displayed improved healing at 2 and 4-week postprocedure. Treatment with AMP cells appears to provide the greatest effect at the 4-week time point. It is theorized that this is because the cells provide a continuous supply of the necessary secreted factors, while the ACCS is essentially a single-dose delivery. Future experiments will test sustained-release delivery of ACCS and should include later time points to determine whether improvement in tendon material properties continues. AMP cells–treated tendons demonstrated a more rapid healing rate and restoration of normal material properties, especially with respect to Young modulus and yield strength.

## Figures and Tables

**Table 1:** Summary of mechanical properties

	Week 1, *M*±SD		
	Control	ACCS	AMP cells
Ultimate tensile strength, MPa	2.75±1.84	3.19±2.6	1.47±0.71
Young modulus, MPa	0.14±0.06	0.08±0.02	0.06±0.05
Breaking strength, N	21.3±10.7	19.5±6.33	10.4±6.48
Stiffness, N/mm	13.57±6.75	14.88±5.28	9.67±5.69
	Week 2, *M*±SD		
	Control	ACCS	AMP cells
Ultimate tensile strength, MPa	2.9±0.54	2.9±0.72	4.2±1.68
Young modulus, MPa	0.06±0.02	0.06±0.02	0.12±0.08
Breaking strength, N	38.2±4.29	28.9±10.13	37.35±6.5
Yield, MPa	1.55±0.35	1.97±0.41	2.39±1.33
Stiffness, N/mm	15.5±0.71	11.57±4.32	17.78±6.59
Cross-sectional area, mm^2^	3.49±0.44	5.72±2.03	4.64±1.65
	Week 4, *M*±SD		
	Control	ACCS	AMP cells
Ultimate tensile strength, MPa	4.78±1.63	5.89±1.12	7.16±2.16
Young modulus, MPa	0.09±0.04	0.12±0.02	0.25±0.02
Breaking strength, N	39.98±11.68	45.64±3.89	41.39±7.13
Yield, MPa	2.93±0.54	3.14±0.96	4.2±0.83
Stiffness, N/mm	24.71±5.5	21.63±6.48	27.5±4.81
Cross-sectional area, mm^2^	3.44±1.63	4.78±1.86	8.93±1.93

ACCS indicates amnion-derived cell cytokine solution; AMP, amnion-derived multipotent progenitor; SD, standard deviation.
